# Think like a Virus: Toward Improving Nanovaccine Development against SARS-CoV-2

**DOI:** 10.3390/v14071553

**Published:** 2022-07-15

**Authors:** Nura A. Mohamed, Haissam Abou-Saleh, Hana A. Mohamed, Mohammad A. Al-Ghouti, Sergio Crovella, Luisa Zupin

**Affiliations:** 1 Biomedical Research Center (BRC), Qatar University, Doha P.O. Box 2713, Qatar; nura.adam@qu.edu.qa (N.A.M.); hasaleh@qu.edu.qa (H.A.-S.); 2 Biological Science Program, Department of Biological and Environmental Sciences, College of Arts and Sciences, Qatar University, Doha P.O. Box 2713, Qatar; hanaadam3@gmail.com (H.A.M.); sgrovella@qu.edu.qa (S.C.); 3 Environmental Science Program, Department of Biological and Environmental Sciences, College of Arts and Sciences, Qatar University, Doha P.O. Box 2713, Qatar; mohammad.alghouti@qu.edu.qa; 4 Institute for Maternal and Child Health IRCCS Burlo Garofolo, 34137 Trieste, Italy

**Keywords:** nanotechnology, vaccine, SARS-CoV-2

## Abstract

There is no doubt that infectious diseases present global impact on the economy, society, health, mental state, and even political aspects, causing a long-lasting dent, and the situation will surely worsen if and when the viral spread becomes out of control, as seen during the still ongoing coronavirus disease 2019 (COVID-19) pandemic. Despite the considerable achievements made in viral prevention and treatment, there are still significant challenges that can be overcome through careful understanding of the viral mechanism of action to establish common ground for innovating new preventative and treatment strategies. Viruses can be regarded as devil nanomachines, and one innovative approach to face and stop the spread of viral infections is the development of nanoparticles that can act similar to them as drug/vaccine carriers. Moreover, we can use the properties that different viruses have in designing nanoparticles that reassemble the virus conformational structures but that do not present the detrimental threats to human health that native viruses possess. This review discusses the current preventative strategies (i.e., vaccination) used in facing viral infections and the associated limitations, highlighting the importance of innovating new approaches to face viral infectious diseases and discussing the current nanoapplications in vaccine development and the challenges that still face the nanovaccine field.

## 1. Introduction

Despite the considerable achievements made in the field of infectious diseases, particularly in the context of viral infections, viruses are still responsible for a wide number of global hospitalizations and death, with a disease burden of over 420 million infection cases in 2019, prior to the coronavirus disease 2019 (COVID-19) pandemic period [1]. In addition, the effectiveness of synthetic agents (anti-viral, vaccines) designed for the treatment or prevention of the infectious diseases is still limited by many challenges [2,3,4], including severe systemic side effects, drug tolerance, and drug resistance, which in light of the most recent pandemic, presents a global health issue. Furthermore, the rapid emergence of new viruses through evolution makes the situation even more complex [5,6]. With scientists focusing on discovering new antiviruses and vaccines, we and others believe that more effort should be made toward finding creative strategies that will unleash the maximum efficacy of the currently available antivirals and vaccines [7,8]. Furthermore, these strategies can be employed to overcome the safety concerns associated with the use of the different types of vaccines. Consequently, new vaccines can be developed for infectious diseases using these strategies, which will be the focus of this review. 

**Figure 1:**
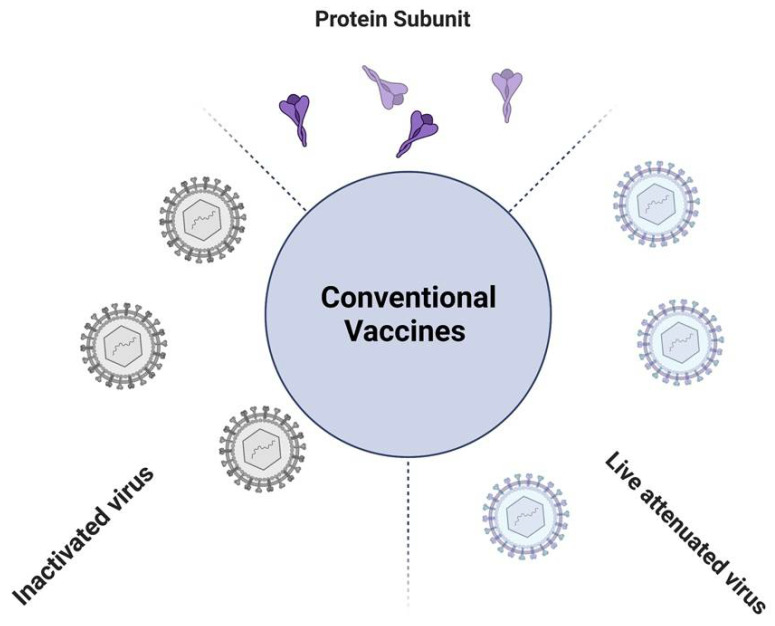
Illustrative figure of the currently available conventional vaccine types.

Vaccination is the most cost-effective strategy to control viral pathogenicity and transmission, thus counteracting infectious diseases. Vaccinations have saved and are currently saving millions of lives, having reduced the mortality rate in pediatric ages by 60% from 2000 to 2019 (from 9.92 to 5.30 million), although in 2019, vaccine-preventable deaths represented 22% of the under-5 deaths worldwide [9]. Conventional vaccines are often based on: (i) attenuated viruses, (ii) killed pathogens (inactivated), and/or (iii) subunits of protein antigens, which will then elicit specific immune responses (Figure 1). 

Even though these vaccines have allowed for the prevention, or the control, of several viruses such as rubella, yellow fever, polio, and measles, and even eradicated some viruses such as smallpox [10,11], some vaccines still require safety and efficacy improvement measures. Thus, the vaccine formulation strategy has turned to different technologies, such as the split-product and cell culture subunit vaccines, where the virions are disassembled and the viral component purified, or the recombinant subunit vaccine, where the antigen protein is produced through recombinant DNA technology in expression vectors as mammalian and bacterial cell cultures [12]. Although the subunit vaccines are safe, they present some drawbacks such as low immunogenicity and failure in generating a solid and efficient immune response by the host. Especially in individuals in fragile periods of life, such as younger children (below 5 years old) and in the elderly, when the vaccination fails to generate an efficient immunization, these subjects are more susceptible to infections [13]. To overcome this issue, adjuvants can be added to the vaccine formulation, improving the level of immunization; currently, aluminum salt-based adjuvants are the most employed type [14].

In addition to that, we still need to overcome the antigenic drift, which may lead to the development of “universal vaccines”, with more formulations inducing broad-spectrum immunity being investigated. Taking this into perspective to the still ongoing pandemic, COVID-19 has killed over 6.16 million persons thus far, it was not until very recently that several universities/companies started working on developing a pan-coronavirus vaccine [15,16]. As an example, the USA army started developing a pan-coronavirus vaccine showing efficacy against both SARS-CoV-1 and SARS-CoV-2 (and its variants of concern—VOC) that is now in phase I human trial (ClinicalTrials.gov Identifier: NCT04784767) [17]. However, older viruses, such as the influenza virus, have been responsible for a considerable number of deaths as well. Merely one century ago, it was evaluated that the Spanish flu killed 20–40 million persons in 1918, and two less severe pandemics occurred in 1958 and 1968 [18]; actually, the World Health Organization (WHO) estimates the annual death burden of influenza to be 250,000–500,000 deaths globally. The death rate could be higher, especially with the influenza-associated respiratory complications and other supervened respiratory infections related to the influenza virus [19]. Such considerable death rates across new emerging viruses and the dreadful ones that have been there for decades requires careful thinking about how we study and handle viral infections. Looking through the literature, we can find that considerable amounts of attempts and studies focused on developing innovative prevention and treatment strategies for different viral infections. These attempts can be linked together to find common grounds and shared perspectives to build a unique platform that could be used to face new viruses in the future.

## 2. Challenges of Vaccine Development

In spite of the recent progress in the development of conventional vaccines, further, improvement is still required, especially to increase the immunogenicity and reduce toxicity, instability, and side effect issues [20]. Additionally, synthesizing conventional vaccines is quite expensive, and the yield is often meager. Vaccine development is very challenging, with the most notorious hurdles seen during the transition from the laboratory to the clinical trial-enabling activities. Such hurdles include (i) vaccine synthesis, (ii) determining optimum concentration, (iii) studying pharmacokinetics/pharmacodynamics of the synthesized vaccine, (iv) manufacturing process complexity, and (v) difficulty in optimizing the analytical and clinical assays. Additionally, it is important to keep in mind that vaccines are not simple laboratory-synthesized chemical moieties; instead, they are designed to induce a protective immune response in healthy individuals, thus, requiring heavy synthesis and concentration assessments followed by pre-clinical assessments in biological systems that are close enough to the human’s body. A process that could take several years before receiving regulatory approvals for use in clinical trials [21].

Of the different conventional-vaccine types, the live-attenuated pathogens present a low risk of conversion to pathogenic virulence, while inactivated vaccines may be associated with weak immune responses [21]. Current conventional techniques used in the vaccine development field have reached their limits, which is where nanomedicine comes in handy to improve the efficacy of the current conventional formulation. Many studies suggest that incorporating nanoparticles in the vaccine synthesis can elicit a more vigorous immune response [22,23]. Many nanoparticles present immunomodulatory effects and can function as both delivery systems, and immunostimulatory adjuvant [24,25,26], in addition to acting alone as immune potentiators, enabling them to overcome the current limitations associated with the traditional adjuvants. Moreover, nanoparticles offer controlled and sustained release, thereby extending the half-life of the loaded vaccines [27]. A new generation of nanoparticle-based vaccines that promote enhanced antigen presentation and strong immunogenicity are being studied to overcome the limitations of the conventional and subunit vaccines [28]. These nanoparticles are synthesized from either biological or synthetic sources. For this purpose, different engineered nanoparticles have been developed thus far, including lipids, liposomes, emulsions, polymeric nanoparticles, inorganic nanoparticles, immunostimulatory complexes (ISCOMs), virus-like particles (VLPs), and metal and nonmetal organic and inorganic nanoparticles [28,29]. Despite the many physicochemical properties engineered nanoparticles have, they are still associated with some issues that need to be improved. Nanoparticles’ composition, size, morphology, charge, hydrophobicity, and route of exposure are significant aspects that elicit the nanoparticles’ toxicity and immune responses. Therefore, physicochemical characteristics and nanotoxicology need to be thoroughly investigated before considering nanoparticles to be used as vaccine carriers [30].

Nanoparticles provide an option of different routes of administration, as some of them can be administered subcutaneously, and others are given through intramuscular injections or the mucosal sites (intranasal, oral, and sublingual) [31,32,33]. Recent progress in nanomedicine has paved the way for preparing nanoparticles with unique physicochemical properties, with many parameters such as size, shape, surface chemistry, hydrophilicity, and solubility being tuned and designed, allowing for the possibility of tailoring them for different biological purposes [34]. Furthermore, depending on the nanoparticles’ physiochemical properties, they could be used to incorporate single or multiple molecules, including antigens and nucleic acids, making them highly interesting in vaccinology. Nanoparticles either exist naturally in the environment or can be engineered in the laboratory [35]. Engineered nanoparticles have more to offer, as they can be designed to either target or avoid interactions with the immune system. These attractive properties have led to the development of the nanovaccine field, where vaccines are incorporated into the nanoparticles through: (i) conjugation (covalent functionalization), (ii) encapsulation (physical entrapment within the nanoparticles), and (iii) adsorption (on the surface of the nanoparticles) [29], which protect the native antigen from proteolytic degradation and/or improve antigen delivery to antigen-presenting cells (APCs) [36]. The APC system plays an important role in innate and adaptive responses, and it consists of professional and nonprofessional cells. Professional APCs include B-cells, macrophages, and dendritic cells (DCs), and these cells present antigens to helper T cells. Conversely, non-professional APCs, including thymic epithelial cells and vascular endothelial cells, function as antigen presenters only in specific circumstances and for brief periods [37,38].

## 3. Toward Overcoming Challenges Facing Vaccine Development: Think like a Virus

However, why rediscover and design nanoparticles to be used as vaccine carriers to face the natures’ devil nanoparticles (viruses)? Instead, we need to think and act like viruses and use the various attractive properties viruses provide in designing viral-mimicking nanoparticles. Different viruses have different affinities to different cells, tissues, organs, age susceptibility, the environment, and other criteria that we can use to achieve selective and targeted delivery. For these reasons, it might be ideal to use viral shells as nanocarriers for the development of nanovaccines. The most toxic and dangerous part of the virus is the genetic component inside the shell, but if we studied the different viral shells that have been around us for years, while designing synthetic structures that mimic them, we can end up having different nanocarriers with different viral mimicking properties. Viruses have many attractive properties that can be used in designing nanodrugs and nanovaccines: immune system evasion, physiochemical characteristics, biodistribution, tissue tropism, specific high-affinity receptors, cell entry, and endosomal escape. A quick literature screening shows the progress of the nanoformulation-based vaccines status during the past few decades. A PubMed search of the keywords nanoparticles and vaccines returns 5850 results, with only 70 articles before 2003 and 5780 after that. Furthermore, the year 2021 showed the highest amount of publications in this area, with 1136 returns. Thus far, there are different types of nanoparticle-based vaccines approved for human use, with SARS-CoV-2 vaccines being the most recent ones. Therefore, later in this review, we focus on the SARS-CoV-2 nanovaccines. The huge jump in the nanovaccines scientific research and the gathered information can be used to find common ground and shared perspectives to build a unique platform to help face new viruses in the future.

**Figure 2:**
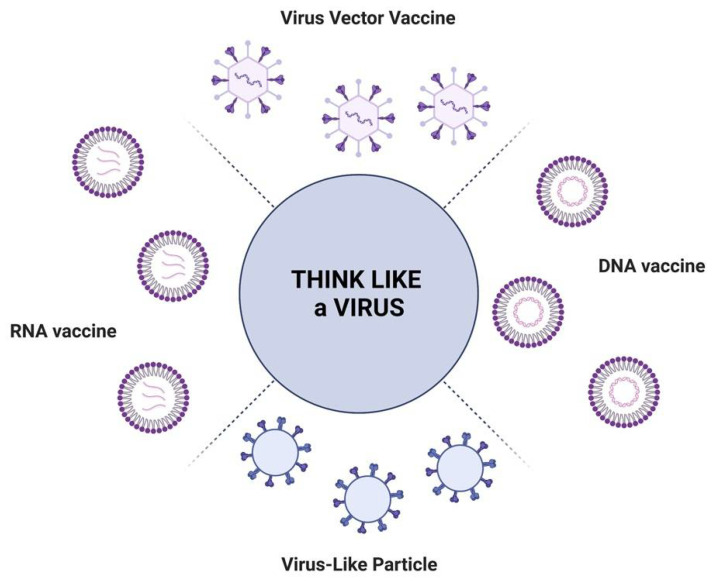
Illustration of the different vaccine strategies exploiting nanoformulations.

Currently, the vaccines exploiting nanoformulations include nucleic acid vaccines encapsulated in lipid-based nanoparticles, virus-like particle (VLP) vaccines, and viral vector vaccines (VVV) (Figure 2).

## 4. Examples of Current Nanoformulation-Based Vaccines

### 4.1. Nucleic Acid Vaccines Encapsulated in Lipid-Based Nanoparticles—RNA and DNA Vaccines

Among the nucleic acid vaccines, RNA vaccines are the most promising approach and can be divided into two main categories: non-replicating mRNA and self-amplifying mRNA (saRNA). mRNA vaccines are produced from linear DNA, resembling fully processed mRNA (open reading frame flanked by UTR, with a 5′ cap and poly (A) tail). Some chemical modifications to the nucleotide can be used to improve the stability of the mRNA molecule [39]. SaRNA vaccines are genetically engineered nucleic acids derived from self-replicating single-stranded RNA viruses lacking the capability to produce infectious virions. Only the genetic material is able to replicate, further incrementing the yield of intracellular RNA for protein translation. Therefore, the vaccine can be inoculated in a single dose and at lower concentration [40]. mRNAs are usually processed within the cytosol, where they can be directly translated into proteins [39]. Animal studies have shown that after injection into the muscle, mRNA molecules are engulfed by APC cells, such as DC and monocytes and are delivered to the draining lymph node [41]. Additionally, it has been observed that mRNA vaccines can present direct passive trafficking toward lymph nodes, where B lymphocytes are able to capture the lipidic nanoparticle, although this route appears to be less frequent with respect to cellular transport through APCs [42]. In the lymph nodes, lymphocytes type B will interact with the antigens presented by APCs and activate the following immunological cascade, resulting in type B differentiation and antibodies production [43]. Moreover, foreign mRNA inside the cells is also sensed by pattern recognition receptors such as toll-like receptors 3, 7, and 8 and by retinoic-acid-inducible gene I (RIG-I) and melanoma differentiation-associated 5 (MDA5), which in turn stimulate type I interferon response [44].

Until now, the major drawback of mRNA vaccines has been related to their stability limitations both during and after injection into the host. Nevertheless, great advancements were made with lipid-based nanoformulations that preserve mRNA integrity and at the same time enhance the efficiency of their delivery in host cells. Moreover, mRNA construct optimization has also been introduced to improve nucleic acid durability [45]. The mRNA vaccine can elicit a strong immune response, an advantage and disadvantage at the same time. Besides immunization against the infectious agent, in a minority of the cases it may lead to an exaggerated immune reaction and adverse effects [46]. However, in the recent pandemic, mRNA vaccines have shown great efficacy in terms of immunogenicity, although multiple doses are needed to maintain that immunity, with the EMA and ECDC recommending a fourth dose for mRNA vaccines in fragile subjects and elderly [47].

The other nucleic acid vaccine type is the DNA vaccine, which is based on a plasmid vector where the gene of interest is inserted. Once internalized by the cells, DNA will be transcribed to RNA and transduced to protein. However, while RNA vaccines are processed in the cytoplasm, DNA vaccines need to be transported to the nucleus to be functional [48,49]. A major issue with DNA vaccines is the route of administration. To efficiently enter into the nucleus, DNA vaccines need the employment of specific delivery systems to obtain a high efficiency of transfection [45]. Physical methods include jet injectors, gene gun delivery, electroporation, and microneedle array-based delivery; other techniques use liposomes, virosomes, and other synthetic and natural nanoparticles, exploiting endocytosis by host cells [50]. Immunogenicity is generally lower with respect to those of mRNA vaccines, requiring multiple doses and the contemporaneous presence of adjuvants. An adverse effect to be taken into consideration is the generation of anti-DNA antibodies [46]. The great benefits of nucleic acid vaccines include non-residual infectivity, the avoidance of anti-vector immunity and their expression of residing in the host cells not needing microbial molecules for transcription and transduction. Moreover, they present a low cost of production, high scalability, rapid development from the design to the manufacturing stage, and the adaptation to serve the new emerging variants of the target microorganisms [51].

Effective and stable delivery systems are required for the safe delivery of mRNA and DNA to ensure its in vivo function. Lipid nanoparticles were shown to protect nucleic acid from degradation and maintain cellular uptake and nucleic acid release. The potential of lipid-based nanoparticles as drug delivery systems was recognized almost immediately after their discovery [52]. Lipid-based nanoparticles, particularly liposomes, were first discovered in 1965, while mRNA was discovered in 1961, with the first mRNA-liposome nanoformulation being prepared in 1993 to be used in the influenza vaccine [52]. Moreover, lipid-based nanoparticle–mRNA vaccines have been thoroughly investigated and successfully entered into clinical trials [52]. Additionally, they recently marked a critical milestone for mRNA therapeutics when they were used to face the COVID-19 pandemic. The progress made since the 1960s in both mRNA technologies and lipid nanoparticle-based delivery systems allowed for the rapid development of the current COVID-19 vaccines. During the process of finding the optimum carrier for the COVID-19 vaccine, a variety of lipid nanoparticles were explored and optimized until the current ones were selected, which resulted in the generation of valuable information about different types of lipid nanoparticles that could be used for future improvement and design of different lipid-mRNA based nano-therapeutics [52].

The four different lipid components thoroughly investigated and used for the COVID-19 vaccines were: a neutral lipid distearoylphosphatidylcholine (DSPC), cholesterol, a polyethylene glycol (PEG)–lipid and an ionizable cationic lipid. Previous studies showed that both the neutral lipid and cholesterol provide the lipid nanoparticle with bilayer stability and fusogenic properties [7,8]. PEG–lipids are used to control particle size and to prevent particle fusion and aggregation [9]. Finally, the most important component of the mRNA–lipid vaccine is the ionizable lipids that are used to: (i) enhance the effective and high encapsulation of the negatively charged mRNA, (ii) maintain the neutral charge of the nanoparticle at physiological pH, and (iii) reduce the cationic charge associated toxicity that prolongs the nanoparticle’s circulation lifetime [53]. Noteworthy, ionizable cationic lipids are considered the most promising delivery vectors for DNA, as they form lipid/DNA complexes. Such complex formations protect the DNA content from being digested by nucleases in addition to their ability to cross the cellular membrane and access the cytoplasm, which can be used for macrophage targeting as well as activating different elements of the immune system [54]. Furthermore, lipid nanoparticles can improve the delivery efficacy of mRNA and DNA by rationally designing and modulating lipids to increase their cellular uptake and to enhance their endosomal escape to achieve organ-specific and cell-specific delivery, which can be further explored and used for targeted drug delivery [52]. However, lipid nanoparticles are accompanied by some drawbacks that may limit their use. Such drawbacks include side effects and toxicity concerns, scale-up challenges for sterile production, size control challenges [55], the possibility that the mRNA and DNA might affect the lipid structures, and the thermal sensitivity of such complexes requiring specific storage temperature [56]. In addition, unfunctionalized or unmodified lipid nanoparticles could have significant limitations, including short blood half-life, lack of targeting selectivity, and instability in vivo. Such shortcomings can be overcome by improving the lipid nanoparticle formulations and designs, in addition to choosing the optimum lipid nanoparticle, as different lipids have varying advantages and disadvantages [55].

### 4.2. Virus-like Particle (VLP)

Nanoparticles with optimized viral infection machinery often incorporate virus peptides or proteins into their composition, called VLPs [57]. In 1986, Smith et.al [58] discovered the precipitation of particles presenting antigenic sites on the surface of the sera of patients with leukemia, Down’s syndrome, and hepatitis. However, the biological nature of these particles remained unknown [58] until the late 1970s and early 1980s, when the concept of VLP-based vaccines was introduced; however, it was not until 1989 that the first VLP was approved, which was Engerix-B (HBV vaccine) [59]. VLPs are 20–200 nm self-assembled nanostructures composed of viral proteins (capsid, envelope or core viral proteins), but they lack the genetic materials hindering them non-infectious. VLPs can be formed of single, double, or triple layers and can present one or multiple proteins. Moreover, they can be enveloped or non-enveloped, thus reflecting the virion shape [60]. Their unique targeting properties make them suitable carriers for the delivery of small drugs, proteins, peptides, and genes [61]. Currently, recombinant viral proteins used in the synthesis of VLPs can be expressed in the laboratory using a wide range of expression systems, including plants, yeast, baculovirus/insect cells, prokaryotic cells, mammalian (i.e., CHO, BHK.21, HEK293, CAP-T, Vero 9, ELL-o) and avian cell lines [62,63]. Once prepared, they are then purified from the cellular components; however, it is hard to guarantee that the yield will be 100% free of any cellular components. Therefore, cell-free protein synthesis techniques have been developed to express and produce VLPs in vitro [63]. Usually, VLPs are synthesized by assembling proteins taken from a single virus type; however, they can also be created using the structural proteins from different types of viruses (i.e., chimeric VLPs). Different structural viral proteins from human immunodeficiency virus (HIV), adeno-associated virus, hepatitis B virus (HBV), hepatitis C virus (HCV) and bacteriophages have been previously employed to create VLP. Furthermore, VLPs can be incorporated with other nanomaterials such as lipids; such incorporation enhances viral binding and uptake by the target cells, increasing the encapsulated drug’s payload in the cytoplasm. Furthermore, VLPs are synthesized through the conjugation of the viruses’ functional components, including the host cell’s receptor-recognition domain of the viral protein [57].

VLPs present the advantages of eliciting a strong immune response due to their intrinsic viral structures, while avoiding the risk of replication, as they lack the viral genome, making them particularly safe. Therefore, many VLPs can spontaneously stimulate the immune system, although some adjuvants and immunomodulators can be inserted into the formulation [60]. The first VLP–based vaccine formulations were two anti-HBV vaccines; they were composed of self-assembled HBsAg and were approved for human use more than 30 years ago by the FDA and 20 years ago by the EMA. Then, in the 2000s, the self-assembled HPV L1 capsid protein was approved against human papillomavirus (HPV) infection [60]. VLPs have unique advantages that favor them from other nanoparticles. Such advantages include: (i) their ability to morphologically resemble the structure of the parent virus, (ii) their retention of the parent virus’s immunogenic surface structure, (iii) their retention of the parent virus’s cell uptake and immune processing pathways, (iv) possessing a variety of shapes and structures, and finally (v) being devoid of an intact virus genome. Moreover, VLPs themselves are nonpathogenic and incapable of replication or infection, which increase their safety profile [64,65]. The ability of the VLP to elicit the parent virus’s immune response while having a high safety profile is unique and makes them one of the state of the art nanoparticles to be used as vaccine carriers. However, similarly to the other nanoparticles, VLPs are accompanied with drawbacks associated with design, formulation, purification, stability, storage, immunogenicity, and clinical vaccine efficacy [64]. The fact that VLPs do not have the viral genome affects their stability during the processing steps (e.g., synthesis, purification) and in vivo, which is why VLPs are further modified by coating or enveloping them with other materials (e.g., proteins, polymers, lipids). Additionally, the expression levels of VLPs vary, as budding from the cell membrane is a key step for VLP formation. This step is also associated with many impurities from the host cell, including cell debris, proteins, DNA, and lipids that represent a great challenge, as it can be hard to completely purify the VLP from all these impurities [65].

### 4.3. Viral Vector Vaccines (VVV)

VVVs exploit non-pathogenic and harmless genetically engineered viruses. The viral genome is modified by inserting the gene of interest for the expression (generally a structural protein from another virus), and the gene related to virulence is removed, rendering the virus replication incompetent or less virulent [66]. A major limitation for VVVs is the need for another serotype in the booster and the pre-existing immunity concerns [67,68]. In the early 1990s, a new generation of recombinant and DNA-based vaccines was developed; however, these vaccines were associated with many disadvantages, including the activation of oncogenes, having low immunogenicity, and elicitation of anti-DNA antibodies [69,70]. Different types of viral vectors are currently exploited, poxvirus and adenovirus being the most employed. Other vectors include adeno-associated virus, retrovirus, lentivirus, cytomegalovirus, and sendai virus [68]. Preparing nanoparticles that contain both synthetic and viral components led to the establishment of a novel class of delivery materials. This class combines both the virus and functional synthetic material properties [57]. For instance, in 2011, Kostiainen et al. [71] successfully synthesized nanoparticles consisting of temperature-switchable polymers that were fabricated with chlorotic mottle virus (CCMV) [71]. The CCMV modified the synthetic material’s physical properties, as the synthesized nanoparticle displayed temperature-dependent assembly/disassembly properties [57]. The viral structure was tightly linked to their biological function, as it protected the genome in the inner space of the virion from exogenous degradation. The outer layer of the virions displayed unique surface proteins that helped the virus to identify and target the host cells. Once identified, these proteins interacted with the receptors presented on the host cell membrane, inducing the release of the inner core inside the cell, which was followed by the successful transportation of the genome to the host’s nucleus. This unique structural hierarchy used by viruses could be further utilized in developing a rational strategy to be used for intracellular drug delivery. These nanoparticles (virus-like nanoparticles) could consist of a “core-shell” structure prepared from non-viral components, while the outer shell consists of viral components including proteins specific to the desired host cell [57]. Viral-based nanoparticles are a highly promising field that offers a wide range of smart delivery systems that could be activated to release their inner cargo when subjected to stimuli such as changes in temperature, pH, as well as other physical and chemical conditions. Bacteriophages and plant-based viruses are usually favored over mammalian viruses, as they cannot proliferate in humans, minimizing the risk of triggering undesired side effects. Non-human enveloped virus nanoparticles are a composite of two parts: the first part is the empty capsid, an outer protein shell that makes the nanoparticle resemble the native virus in morphology, and the loaded material. The unique structure of the viral-based nanoparticles, in addition to their ability to disassemble under certain conditions, offers a convenient yet powerful targeted drug delivery strategy [72].

Moreover, viral-based delivery systems are remarkable novel carriers that can efficiently encapsulate and deliver the loaded cargo. However, they usually require a host for the production and purification procedures, which may come at a high cost. Moreover, these nanoparticles have a higher risk of triggering immune responses. Hence, these two challenges make it essential for developing tuning techniques that can enhance the nanoparticle’s stability, immunomodulatory effect, targeting specificity, and thus localization properties [73]. Such tuning techniques involve the incorporation of materials in the synthesis of the viral nanoparticles. The incorporation will subsequently improve the nanoparticle properties that stem from its size, shape, and composition to the viral properties (e.g., targeting proteins) affecting the type of cell targeted, the mode of cellular uptake, and intracellular trafficking. Moreover, existing nanoparticles can be coated with viral proteins to mimic the virus. Additionally, it is crucial to study the nanoparticles’ uptake, escape, and trafficking mechanism, as these mechanisms often involve different signaling cascades that can affect the cells/organs or tissues in the long term [73].

Currently, there are many viral vectors to choose from for VVV development; therefore, when designing a VVV for a specific infection, the handling of viral vectors, the preferred route of administration, and safety should all be considered [74]. Using VVV for viral infections is beneficial, as it is a well-established technology, it offers strong immune response that involves B cells and T cells, and it does not require adjuvants. However, this technology has some drawbacks and faces other challenges; for example, previous exposure to the vector could reduce effectiveness, minimizing its ability to produce the required immune response. Despite the fact that the commercial manufacturing process of viral vectors had been developed decades ago, the synthesis of VVV requires product optimization in order to fulfill the requirements of quality and cost-efficiency. Such requirements are due to the complexity of viruses, which makes manufacturing the second main challenge associated with VVV development. VVV requires living cell lines that contain the missing components to replicate, which is achieved using adherent cell system or cells grown in suspension. Both ways are complicated, as they are associated with the difficulty of maintaining good balance between high recovery yield, impurity clearance and the reduction of cost [75].

Table 1 describes some examples of viral nano-based vaccines already employed in different clinical trial stages.

Table 2 describes potential nanoformulations to be used as viral antigen carriers.

## 5. Immune Activation by Nanovaccines

Both humoral and cell-mediated immune responses can be evoked by the nanoparticle’s physiochemical characteristics, as nanoparticles are recognized by the antigen-presenting cell (APC) system [37,38]. Furthermore, it has been shown that nanoparticle characteristics can affect vaccine efficacy, as they can influence the binding of the nanoparticles to the cell surface and its entry into the cell (i.e., cellular uptake). Furthermore, some NPs can maximize the immune response, as they can provide a 30-times increase in cellular uptake compared to the vaccine alone. Many factors can affect the NPs’ cellular uptake, with the most important ones being the nanoparticle’s size, shape, and surface charge [76,77,78]. Nanoparticle size and distribution greatly influence its interaction with the cell membrane, therefore affecting the nanoparticle’s entry through physiological barriers, with different cell lines allowing specific nanoparticle sizes and shapes to pass through their membranes [79,80]. More importantly, nanoparticle size plays a decisive role in determining the type of immune response elicited (e.g., humoral or cellular immune systems). For instance, studies showed that nanoparticles that are smaller than 500 nm stimulate the responses of the CD4^+^ Type 1 and T CD8^+^ cells, whereas nanoparticles with sizes larger than 500 nm often stimulate T CD4^+^ Type 2 cells that produce antibodies [80,81,82]. Moreover, other studies showed that nanoparticles larger than 1000 nm are internalized by phagocytosis, while nanoparticles less than 1000 nm are often internalized by micropinocytosis or endocytosis (clathrin dependent/independent or caveolin independent) [83]. Smaller nanoparticles are taken up more efficiently by the DCs and are transported to the lymph nodes, which is why they are considered better for drug delivery; however, an universal correlation between nanoparticle size and host response has yet to be achieved [84]. Nanoparticle shape also influences its cellular uptake, with some studies showing that spherical nanoparticles are taken up more than the rod-shaped and elliptical ones [85]. This is due to the fact that spherical nanoparticles have higher phagocytosis efficacy compared to other shapes. As a result, it is important to take nanoparticle shape into consideration regarding the production of nanovaccines [86,87].

NP composition also influences the nanovaccine’s efficacy, with metallic nanoparticles (i.e., nickel, iron and gold) inducing a humoral response and promoting host cell recruitment and the activation of APC and cytokine production [88]. The loading type used for the nanoparticles also influences the functionality of the nanovaccine. For example, the loading of the antigens inside the nanoparticles protects the cargo from its exposure to the external environment and its degradation, while its incorporation within the cells may bio-mimic the pathogens’ antigen features [89]. Moreover, it is important to consider the nanoparticle’s aggregation level in the blood when designing nanovaccines, with the nanoparticle’s surface charge being the determining factor of the aggregation level. In addition, nanoparticle surface charge affects cellular uptake, as it influences the nanoparticle’s interactions with nonspecific and undesired cells that have opposite charges. For this matter, cationic nanoparticles are used when negatively charged cells or DNA are the target [90,91,92]. In addition, cationic nanoparticles are known to be internalized more rapidly by DCs than other types of nanoparticles [93].

The use of the viral-specific antigens in designing nanovaccines brings us to multivalent antigen presentation, which is a technique where viral glycoproteins are presented to the immune system to strengthen the response. Such a technique is known to increase the potency of humoral immune responses, as viral glycoproteins behave similarly to pathogen-associated molecular patterns (PAMPs). Furthermore, in this technique, viral glycoproteins, which are specific to each virus, are used to elicit antigen-specific antibodies [94,95,96,97]. For this reason, many vaccines contain glycoproteins that function in training the immune system to better recognize a specific virus. In addition, surface glycoproteins are considered “keys” to enter cells, as they recognize and unlock specific targeted cells to unleash the viral infection. Due to their strong immune response and function, glycoproteins are used in designing nanovaccines. In this approach, a protein scaffold that mimics the shape of the desired virus is used, and then glycoproteins are arranged on the surface of the scaffold to ensure that it triggers a strong immune response. The development of scaffolds that are capable of presenting these glycoproteins has been an ongoing challenge, as it can be difficult to assure the stability of the glycoproteins incorporated into non-protein-based nanoparticles such as polymers, liposomes, lipids, metals [97,98,99,100]. This challenge necessitates the need for developing nanomaterials that can act as scaffolds for glycoprotein incorporation. Ueda et al. [101,102] and Antanasijevic et al. [102,103] developed methods where they can synthesize scaffolds using artificial proteins as natural proteins that do not always display viral glycoproteins. These scaffolds are then further functionalized using viral glycoproteins. Thus far, this approach has been used in synthesizing vaccines for HIV, influenza, and RSV. The results have shown that for each vaccine: (i) viral glycoproteins could attach themselves to the scaffold; (ii) the scaffold–glycoprotein complex then successfully assembled into vaccine particles that mimicked the virus; (iii) the complex strengthened and maximized the immune response [104,105]. These results showed that functionalizing artificial scaffolds using glycoproteins would improve vaccine development and give us better control over vaccine design to develop attractive scaffolds for multivalent antigen presentation [104,105].

### 5.1. Immune Activation of Lipid-Based Nanoparticles—RNA and DNA Vaccines

Animal studies showed that after injection into the muscle, RNA vaccines are engulfed by APC cells, such as DC and monocytes, and are delivered to the draining lymph nodes [41]. Additionally, mRNA vaccines can also present direct passive trafficking toward lymph nodes, where B lymphocytes are able to capture the lipid nanoparticle, although this route appears to be less frequent with respect to cellular transport through APCs [42]. The RNA is then internally processed, transduced into protein products, and degraded by proteasome in antigen peptides that are loaded and presented on the cellular surface through the major histocompatibility complex (MHC). In the lymph nodes, APCs present antigens to lymphocytes type B, activating an immunological cascade that results in type B cell differentiation and antibody production, as well as lymphocyte type T induction and the activation of the antigen specific CD8+ T cells response [43,49]. Moreover, foreign mRNA inside the cells is sensed by pattern recognition receptors such as toll-like receptors 3, 7, and 8 and by retinoic-acid-inducible gene I (RIG-I) and melanoma differentiation-associated 5 (MDA5), which in turn stimulate type I interferon responses [44].

DNA vaccine can activate both cellular and humoral immune responses, with antigen presentation in the DNA vaccines relying on the professional APCs that phagocytize apoptotic bodies from somatic cells, such as keratinocytes and myocytes previously transfected with the plasmid, leading to antigen presentation to both CD4^+^ and CD8^+^ T cells and subsequent activation of the cytotoxic T lymphocyte (CTL) response [49]. Two other routes have been hypothesized, but they may occur less frequently by transfected somatic cells presenting the antigen to CD8^+^ T cells or to B cells (which induced antibody production) or by the direct internalization of the plasmid by APCs [49]. 

### 5.2. Immune Activation of Virus-like Particle (VLP)

VLPs are directly recognized and taken up by dendritic cells through pathogen recognition receptors (PRRs) on the membrane surface, as toll-like receptors (TLR) or C-type lectin receptors (CLRs) that are the same receptors that naturally recognize microorganisms. After the uptake, DCs transfer them to the lymph nodes, where they mature and process the antigens into small peptides. DCs produces pro-inflammatory cytokines such as TNF-α and IL-1β that in turn recruit more APCs and promote antigen proteolysis. Subsequently, DCs present antigens with MHC to CD8^+^ and CD4^+^ T cells, leading to a cytotoxic T lymphocytes (CTL) response and B cell activation, which produces specific antibodies [60,106].

### 5.3. Immune Activation of Viral Vector Vaccines (VVVs)

VVVs themselves, resembling the characteristics of viruses, promote a high immune response. After delivery, VVVs target DCs and macrophages and are sensed by PRRs, such as TLR9 recognizing dsDNA, inducing a type I interferon response. After being delivered to the DCs, VVVs are then drained by the lymph nodes and are presented to the T cell, priming both antigenic and inflammatory signals and leading to inflammatory cytokine production. Therefore, VVVs activate both the CD4+ and CD8+ T cells, leading to the activation of antibody-secreting B cells and CTL response. The production of interferons and pro-inflammatory molecules further boosters the immune response, promoting the generation of long-term immunological memory [67,107].

**Figure 3:**
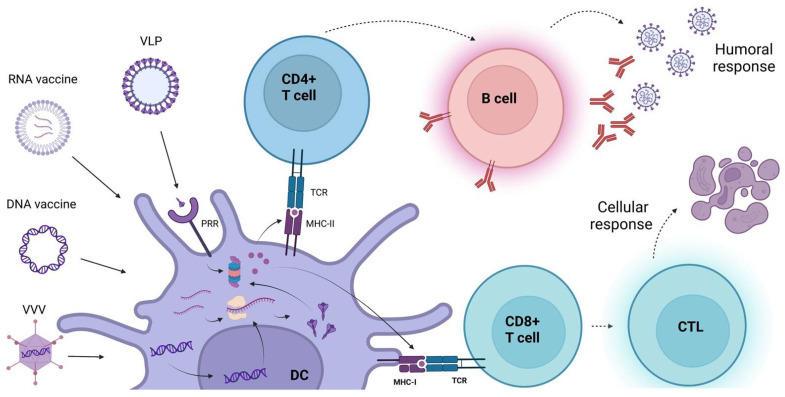
Immune activation induced by nanovaccines. VLP, virus-like particles; VVV, viral vector vaccine; DC, dendritic cells; PRR, pattern recognition receptor; MHC, major histocompatibility complex; TCR, toll-like receptor.

Figure 3 describes the immune activation induced by the different types of nanovaccines.

## 6. Nanovaccines Used in Facing the Most Current Pandemic: SARS-CoV-2

**Figure 4:**
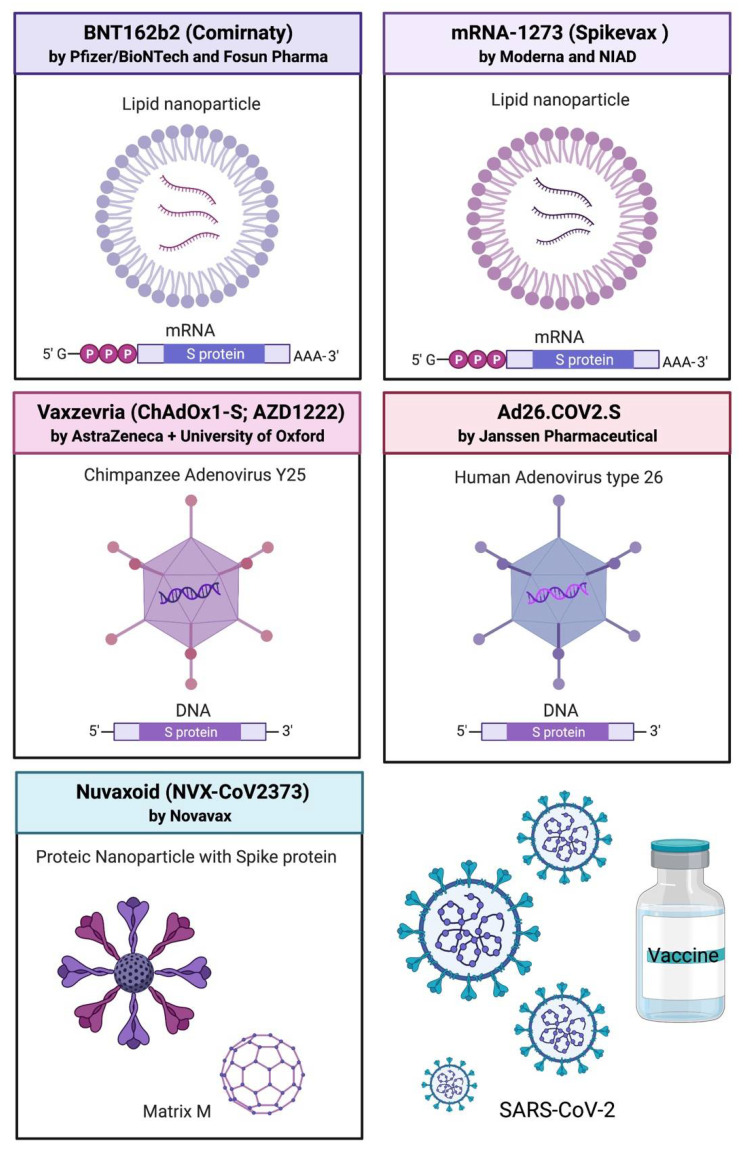
Currently available and approved nanovaccines against SARS-CoV-2.

Severe acute respiratory syndrome coronavirus 2 (SARS-CoV-2) is a beta coronavirus and the etiologic agent of the respiratory coronavirus disease 2019 (COVID-19). SARS-CoV-2 is an enveloped virus, presenting four main structural proteins, namely nucleocapsid (N), which is localized internally; spike (S) and envelope (E) proteins, which are transmembrane proteins, and membrane (M) protein, which is an integral membrane protein [108]. Spike glycoprotein is a trimeric molecule protruding from the virus surface, and it is essential for viral infection. A receptor binding domain (RBD) present in the S1 subunit is required for the binding of the virion with the angiotensin-converting enzyme 2 (ACE2) expressed in the host cells. After attachment, the Type II transmembrane serine protease (TMPRSS2) cleaves S, allowing for fusion between virions and host cells and for virus entry [108]. Based on these characteristics and on the neutralizing antibodies against S induced during COVID-19 natural disease [109], most vaccines are designed to promote immunization against the spike protein. Figure 4 summarize the current available COVID-19 vaccines.

### 6.1. BNT162b2 (3 LNP-mRNAs, Comirnaty) by Pfizer/BioNTech and Fosun Pharma

BNT162b2 is an mRNA-based vaccine encapsulated in lipid nanoparticles encoding for the membrane-anchored SARS-CoV-2 full-length spike (S) protein, modified with two proline mutations to block S in a prefusion conformation [110]. In addition, the mRNA also presents an N1-methyl-pseudouridine base modification for uridines to inhibit innate the immune response and to enhance protein expression [111]. Thirty micrograms of mRNA of BNT162b2 are initially administered in two intramuscular doses [112], but the antibody titers decrease a few months after the last vaccination. Therefore, at the end of 2021, a booster dose was recommended by the WHO [113], as well as a vaccination in the pediatric ages (from 5 years old) [114]. This showed great efficacy, with 91% protection conferred against symptomatic COVID-19. Nevertheless, it has lower efficacy against the Omicron variant (65–75%), with a significant drop after 10 weeks from the booster dose (50%) [115]. The lipid nanoparticle consists of an ionizable lipid, ALC-0315, which presents a pKa of 6.09 and a tertiary amine group. This lipid can switch its charge from neutral to cationic (inside the acidic pH of endosomes). Moreover, ALC-0315 is a biodegradable phospholipid, presenting: distearoyl phosphatidylcholine that mimics cellular membrane lipids; cholesterol that acts by stabilizing the lipid bilayer; ALC-0159 (a PEG2000 lipid) that carries a dialkyl chain of 14 carbon moieties to promote the disaggregation of the lipid nanoparticle [116].

### 6.2. mRNA-1273 (Spikevax, Moderna and National Institute of Allergy and Infectious Diseases (NIAID))

mRNA-1273 is an mRNA vaccine formulated in lipid nanoparticles encoding a transmembrane-anchored SARS-CoV-2 spike protein with two proline modifications that stabilizes the protein in the perfusion conformation and presents the native furin cleavage site [117]. mRNA-1273 is administered in two intramuscular doses. Moreover, a booster dose is recommended [113,118]. It displayed a general efficacy of 94% against any type of COVID-19, being 98% effective in preventing severe disease and 63% effective in preventing asymptomatic infection [119]. The lipid nanoparticles of mRNA-1273 showed a similar composition to BNT162b2, an ionizable lipid presenting a tertiary amine group, the SM-102 (pKa = 6.68), that can switch its charge from neutral to cationic (inside the acidic pH of endosomes). Moreover, they are biodegradable; they also present the distearoylphosphatidylcholine phospholipid, simulating the lipids of the cellular membrane, cholesterol to stabilize the lipid bilayer, where the PEG_2000_-DMG carries a dialkyl chain of 14 carbon moieties for rapid dissociation of the lipid nanoparticle [116].

Lipid nanoparticles are an essential element of both BNT162b2 and mRNA-1273 vaccines, to protect them from RNAse degradation. After injection, the PEG–lipid is dissociated from the lipid nanoparticles, and the nanoparticles can be taken up by the cells; then, in endosomes, the acidic pH protonates the lipid nanoparticles, which changes their structure, disrupting the membrane and releasing the mRNA into the cytosol [116]. Moreover, lipid nanoparticles are too big (~100 nm) to enter directly into blood circulation; thus, they are drained to the lymph nodes, avoiding systemic effects [42]. A problematic issue of BNT162b2 is the stability and storage at −80 °C, requiring strict control of the temperature during transportation and storage until delivery, while mRNA-1273 requires a more affordable temperature of −20 °C [116]. 

### 6.3. Vaxzevria (ChAdOx1-S; AZD1222), by AstraZeneca + University of Oxford

Vaxzevria is a replication-deficient simian adenoviral vector derived from the chimpanzee adenovirus Y25 that encodes for a full-length SARS-CoV-2 S protein. Since their size is around 80–100 nm, they can be acknowledged in the nanotechnology field [120]. The vaccine is produced from a plasmid and a ChAdOx1 vector. The plasmid presents the S protein sequence (2-1273 amino acids) and a tissue plasminogen activator leader sequence localized at 5′ [121]. The plasmid is inserted into the ChAdOx1 DNA bacterial artificial chromosome (BAC) vector through recombinant technology, and then, the adenovirus genome is excised from the BAC, and the virus is amplified in the E1-complementing cell culture. The virus lacks the E1 and E3 sequence, where E1 is necessary for replication, while the loss of E3 increases the length of the exogenous insert to 8 kB [121,122]. Adenoviruses are ds DNA viruses with an icosahedral protein capsid, with more than 50 different serotypes affecting the human population ubiquitously, since most individuals have presented seropositivity for one or more types [120]. Therefore, usually, in vaccines, monkey adenoviruses are employed [120]. The reported efficacy of Vaxzevria is 76% against symptomatic COVID-19 with no severe illness in the vaccinated group. After 20 weeks, there was no vaccine effect against the Omicron variant, but there was a 40% effect against the Delta variant. Nevertheless, a booster mRNA vaccine dose increases immunization at a level equal to three mRNA vaccine doses [115,123].

### 6.4. Ad26.COV2.S by Janssen Pharmaceutical

Ad26.COV2.S is a replication-incompetent viral vector vaccine encoding the prefusion stabilized S immunogen (S.PP) SARS-CoV-2 protein. Ad26 E1-E3-deleted virus derived from the human adenovirus type 26 is employed, while the S.PP immunogen presents a wild-type leader sequence, the full-length membrane-bound Spike protein, furin cleavage site mutation, and two proline-stabilizing mutations [124]. In non-human primates, a single dose of vaccine can induce a potent humoral and cellular response [125]. Although immunization against adenovirus type 26 has been found in the human population, recent findings highlight that it does not suppress the immune response against Ad26 vectors [125,126]. Vaccine efficacy against severe–critical COVID-19 is 77% after 28 days from single intramuscular immunization and 100% after two doses. A booster mRNA vaccine dose was tested in some trials with promising results [127].

### 6.5. SARS-CoV-2 rS/Matrix M1-Adjuvant (Full Length Recombinant SARS CoV-2 Glycoprotein Nanoparticle Vaccine Adjuvanted with Matrix M) NVX-CoV2373, Nuvaxoid by Novavax

Nuvaxoid is a recombinant SARS-CoV-2 spike protein nanoparticle co-formulated with the adjuvant Matrix-M. Nuvaxoid expresses the wild-type full-length SARS–CoV-2 spike glycoprotein, with a modification at the S1/S2 furin cleavage site and two proline substitutions to stabilize the S protein. The purification process from sf9 cells allows S trimers to arrange in ordered protein–protein micellar 27.2 nm nanoparticles with the protein maintained in native conformation [128]. The Matrix-M™ adjuvant consists of thermostable 40 nm nanoparticles, consisting of two different saponin fractions extracted from *Quillaja saponins* Molina bark with cholesterol and phospholipids. It promotes the activation of innate immune cells and antigen processing, enhancing vaccine immunogenicity [128,129]. The vaccine is delivered intramuscularly in two doses with a vaccine efficacy of 90% (in the first trial). Then, in a phase 3 study, when multiple variants are circulating, the vaccine efficacy against moderate–severe COVID-19 reaches 100% [130]. Nuvaxoid has been recently approved by EMA for emergency use [131] but not by the FDA.

In Table 3 the information regarding approved COVID-19 vaccines is presented.

## 7. Ongoing Clinical Trials

As of July 2022, 167 vaccine candidates against SARS-CoV-2 have been tested, and are currently at different clinical trial stages. In Table 4, the number of candidate vaccines employing nanoformulation is reported and classified according to the clinical trial phase and type of platform used. RNA-based vaccines are the most employed nanoformulation with 38 candidate vaccines, highlighting the interest of the pharmaceutical and biotechnological companies in this new form of vaccination. Conversely, traditional vaccines have also been investigated, being 22 vaccines based on the inactivated virus, two on the live-attenuated virus, and 54 on protein subunits (with only 7 presenting nanoformulation, mainly nanoparticles and adjuvated with matrix M).

## 8. Conclusions

As nature’s generated nanoparticles, viruses can inspire scientists to develop sophisticated nanocarriers that will show more efficient cellular targeting, uptake, and internalization properties for the delivery of different cargo materials, including drugs and nucleic acids that can surpass the existing delivery system. Subsequently, their surface proteins can be used to design nanomaterials with stimuli-responsive properties to generate a new class of adjuvants. Nanoparticles possess the intrinsic characteristics to replace old vaccine technologies. Nanoparticles also allow for longer antigen stability, improved immunogenicity, targeted delivery to specific sites, and prolonged release [133]. Different ongoing clinical trials are currently investigating the delivery of nanovaccines through the mucosal surface (oral, nasal and inhalation routes of administration) without the need for intradermal injection, making it a promising approach in developing countries or conditions requiring non-invasive administration routes [134].

Moreover, as current anti-SARS-CoV-2 vaccines have highlighted, nanovaccines present the peculiarity of large-scale production at rapid speed and at relatively low cost. In less than 1 year, different nanovaccines have passed the clinical trial phases and have been delivered to millions of individuals worldwide with optimal safety and immunogenicity profiles, thus being indispensable to counteract the COVID-19 pandemic. The achievement of this goal will lead to the next objective by the Coalition for Epidemic Preparedness Innovations (CEPI): the preparation and approval of a vaccine for the next infectious pandemic agent “X” in 100 days [135]. In this view, nanotechnology should become the way for a fast and soft transition of the nanomedicine platform to and through clinical application.

## Figures and Tables

**Table 1:** Viral nano-based vaccines in different clinical trial stages.

Vaccine Candidate	Infectious Diseases	Type of Nanoformulation	Clinical Trials. Gov Identifier:	Phase
NanoFlu (Quad-NIV)	Influenza A and B	Matrix M nanoparticles	NCT03658629	III
RSV F Particle Vaccine	Respiratory syncytial virus infections	Protein nanoparticle	NCT02624947	III
PepGNP-Dengue	Dengue virus	Dengue virus peptides on a gold nanoparticle	NCT04935801	I
EBV gp350-Ferritin Vaccine	Epstein–Barr Virus	Epstein–Barr virus (EBV) gp350-Ferritin nanoparticle vaccine adjuvanted with Matrix-M1	NCT00106769	I
VRC-HIVDNA016-00-VP, + VRC-HIVADV014-00-VP (boost)	HIV	Experimental multiclade HIV DNA plasmid vaccine, (VRC-HIVDNA016-00-VP), followed by a boost dose of adenovirus-vector vaccine (VRC-HIVADV014-00-VP)	NCT00125970	II
ChAdOx1 MERS	MERS-CoV	Replication-deficient simian adenoviral vector expressing the spike (S) protein of MERS coronavirus	NCT04170829	I
rVSV-ZEBOV-GP (V920 Ebola Vaccine)	Zaire Ebola virus	Recombinant vesicular stomatitis virus with Zaire Ebola virus envelope glycoprotein	NCT02503202	III completed FDA and EMA approval in 2019 (ERVEBO)
Ad26.ZEBOV MVA-BN^®^-Filo	Zaire Ebola virus	Human adenovirus serotype 26 (Ad26) expressing the Ebola virus Mayinga variant glycoprotein, + recombinant, non-replicating modified vaccinia Ankara viral vector encoding glycoprotein from Ebola Zaire (Mayinga), Sudan, and Marburg viruses and nucleoprotein from Taï Forest virus	NCT02543268	III completed EMA approval 2020 (Zabdeno and Mvabea)
V501	HPV	HPV L1 capsid protein, self-assembled into a viral-like particle	NCT00517309	III completed Approved by EMA and FDA 2006 (Gardasil)
HPV-16/18 VLP/AS04	HPV	HPV L1 capsid protein self-assembled into a viral-like particle	NCT00485732	III completed Approved by EMA 2007 and FDA 2009 (Cervarix)
Recombivax HB	HBV	HBsAg self-assembled into a viral-like particle	NCT *	Approved by FDA 1986 and EMA 1999
Engerix B	HBV	HBsAg self-assembled into a viral-like particle	NCT *	Approved by FDA 1986 and EMA 2000

* These trials are old; therefore, their original trial numbers were not found.

**Table 2:** Potential nanoformulations to be used as viral antigen carriers.

Nanoformulation	Antigen	Disease
Chitosan nanoparticles	Antigenic protein	Hepatitis B
Gold nanoparticles	Viral protein	Foot and mouth disease
Gold nanoparticles	Membrane protein	Influenza
Gold nanoparticles	Viral plasmid DNA	HIV
Poly(D,L-lactic-co-glycolic acid) nanospheres	Tetanus toxoid	Tetanus
Poly(D,L-lactic-co-glycolic acid) nanospheres	Hepatitis B surface antigen	Hepatitis B
Alginate-coated chitosan nanoparticle	Hepatitis B surface antigen	Hepatitis B
Chitosan nanoparticles	Live virus vaccine	Newcastle disease
VLPs	Capsid protein	Norwalk virus infection
VLPs	Capsid protein	Norwalk virus infection
VLPs	Influenza virus structural protein	Influenza
VLPs	Nucleocapsid protein	Hepatitis
VLPs	Fusion protein	Human papilloma virus
VLPs	Multiple proteins	Rotavirus
VLPs	Virus proteins	Bluetongue virus
VLPs	Enveloped single protein	HIV
Polypeptide nanoparticles	Viral protein	Coronavirus for severe acute respiratory syndrome (SARS)

**Table 3:** Currently (updated to April 2022) FDA/EMA approved nano-based viral vaccinations against SARS-CoV-2 [132].

Commercial Name	Drug Name and Formulation	Pharmaceutical Company	FDA/EMA Approval Year(s)	Phase
Comirnaty	Nucleoside-modified mRNA BNT162b2, encoding SARS-CoV-2 spike protein encapsulated in lipid nanoparticle	Pfizer-BioNTech COVID-19 Vaccine + Fosun Pharma	EMA 2020 Emergency use authorization FDA approval August 2021	IV NCT04844489
Spikevax	Synthetic mRNA-1273, encoding stabilized prefusion SARS-CoV-2 spike protein encapsulated in lipid nanoparticle	Moderna Therapeutics Inc. and the National Institute of Allergy and Infectious Diseases (NIAID)	EMA 2020 Emergency use authorization FDA approval January 2022	IV NCT04900467
Vaxzevria	ChAdOx1-S Chimpanzee Adenovirus encoding the SARS-CoV-2 Spike glycoprotein	AstraZeneca and University of Oxford	EMA 2021 Emergency use authorization	IV NCT04760132
COVID-19 Vaccine Janssen	Ad26.COV2.S Adenovirus type 26 encoding the SARS-CoV-2 spike glycoprotein	Janssen Pharmaceuticals, Companies of Johnson Johnson	EMA and FDA 2021 Emergency use authorization	IV NCT04817657
Nuvaxoid	SARS-CoV-2 recombinant spike protein formulated in nanoparticles with the adjuvant Matrix-M	Novavax	EMA 2021 Emergency use authorization	III NCT04583995

**Table 4:** SARS-CoV-2 and other virus vaccines employing nanoformulation in clinical trials (updated to 1 July 2022) [132].

Phase	RNA BASED vaccine	DNA Vaccine	Viral Vector (Non Replicating)	Viral Vector (Replicating)	Virus Like Particle	Protein Subunit (Employing Nanoformulation)
**IV**	3		4			1
**III**	3	2	2	1	2	1
**II/III**	4	2	2	2		
**II**	6		2	1	1	
**I/II**	7	5	5	1	2	3
**I**	15	7	7	1	1	2
**total**	38	16	22	6	6	7

## Data Availability

Not applicable.
